# Causes of Emotional Eating and Matched Treatment of Obesity

**DOI:** 10.1007/s11892-018-1000-x

**Published:** 2018-04-25

**Authors:** Tatjana van Strien

**Affiliations:** 1 Behavioural Science Institute, Radboud University Nijmegen, Nijmegen, The Netherlands; 2 Department of Health Sciences, Faculty of Science, Amsterdam Public Health research Institute, Vrije Universiteit Amsterdam, Amsterdam, The Netherlands

**Keywords:** Emotional eating, Obesity, Causes, Depression, DEBQ, Matched treatment

## Abstract

**Purpose of the Review:**

Eating in response to negative emotions (EE) may be an explanatory factor of the weight regain of many dieters. This narrative review presents evidence on possible causes of EE and the association of EE with depression and obesity and discusses implications of these findings for the treatment of obesity.

**Recent Findings:**

Possible causes of EE are high dietary restraint, poor interoceptive awareness, alexithymia, emotion dysregulation and a reversed hypothalamic pituitary adrenal (HPA) stress axis. EE may be the outcome of inadequate parenting or depressive feelings in interaction with genetic susceptibility. There is also robust evidence that EE is a mediator between depression and obesity.

**Summary:**

The association of EE with depression and poor emotion regulation skills suggests that the treatment of obese people with high EE should not focus on calorie-restricted diets but on emotion regulation skills. The DEBQ (Dutch Eating Behavior Questionnaire) enables such a matched treatment of obesity.

## Introduction

Calorie-restricted diets are notoriously ineffective for patients with overweight beyond the short term. In the long term, most of the lost body weight is regained, with some patients even ending up weighing more than before the diet [1, 2]. In community samples, chronic dieting, the tendency to restrict food intake to maintain or lose body weight, has repeatedly been shown to even predict future weight gain (references in [3]). The present narrative review will focus on emotional eating (eating in response to *negative* emotions or stress [4, 5•] as possible explanatory factor of the weight regain of many dieters. The latest results on possible causes of distress-induced emotional eating in terms of mechanisms and etiology will be presented, in addition to recent findings on emotional eating as a mediator between depression and body mass index and weight gain. These findings have implications for the treatment of obesity; therefore, also a framework for matched treatment of obesity will be presented.

### Mechanisms of Emotional Eating

#### ‘Disinhibitor’ of Dietary Restraint

Stress or negative emotions may undermine (disinhibit or release) the self-control of dieters in regard to their self-imposed restriction of food intake. When undereating, the body cannot distinguish self-imposed food restriction from real food shortage and acts as if in the starvation mode: the metabolic rate is slowed down (anabolism) and hunger and appetite are increased [6]. The outcome is that dieters develop intense feelings of deprivation, which make them very vulnerable to abandon their diet [7]. This may particularly hold true for times of stress. Indeed, when put under stress or after induction of negative emotions, dieters showed in various experiments higher food intake compared to non-dieters [8, 9]. Dieting is therefore considered a risk factor for an enhanced tendency towards emotional eating [10, 11]. A further consequence of the continuous struggle of dieters against their hunger sensations may be that they altogether lose contact with their feelings of hunger and satiety which is an additional risk factor for the development of emotional eating [10]. The tendency to eat in response to negative emotions or stress is an a-typical stress response, the typical stress response being not eating because the physiological stress reactions mimic the internal sensations associated with feeding-induced satiety [12] (see for empirical support: [13]).

Emotional eating, as ‘disinhibitor’, requires prior inhibition (i.e. restraint) by definition. It is, however, not yet resolved whether restraint eating is a cause of consequence of emotional eating [14, 15], and this may also differ for various subgroups [16].

#### Emotional Eating as Independent of Dieting

Emotional eating may also occur independent of dieting. As will be elaborated below, it may be the outcome of poor interoceptive awareness, a confusion of internal states of hunger and satiety and physiological symptoms associated with emotions [17], alexithymia or poor emotion regulation strategies. Alternatively, emotional eating has also been associated with a reversed stress response of the HPA (hypothalamic pituitary adrenal) axis (a blunted instead of the typical elevated cortisol response to stress) [18, 19].

##### Poor Interoceptive Awareness, Alexithymia and Emotion Dysregulation

It has been suggested that the unnatural response of emotional eating is acquired [17], as possible outcome of parenting practices that undermine the psychological and emotional development of the child [20]. When the response of the caregiver is continuously inappropriate—be it neglectful, overly protective or even manipulative or hostile—the outcome may be that the child develops poor interoceptive awareness (poor awareness of feelings of hunger and satiety) [17], high degrees of alexithymia (difficulty in identifying feelings and describing those to other people [21]) and difficulties with adequately regulating its emotions [22–26].

Indeed, poor interoceptive awareness and high alexithymia were both positively associated with self-reported emotional eating [16, 26]. Alexithymia additionally moderated the association between stress and food intake, with women with high degrees of alexithymia showing the atypical stress response of eating the same or even a little more after stress (the belief of having to give a speech in front of an evaluative audience) [27]. Poor emotion regulation strategies, such as suppression of emotions or maladaptive coping strategies such as a reliance on emotion-oriented coping and avoidance of stress by distraction, were also shown to be positively associated with emotional eating [28, 29].

##### Reversed HPA Axis

Emotional eating was associated with posttraumatic stress disorder [30] and, in a large population of African Americans (> 1000), adulthood trauma exposure and childhood emotional abuse [31]. In this study [31], both depression and emotion dysregulation acted as mediator between childhood trauma or emotional abuse and emotional eating. A possible underlying mechanism of this type of emotional eating may be a reversed stress response of the HPA axis as possible outcome of chronic stress early in life. Instead of responding to stress with hyper-activation and the typical neurovegetative symptom such as loss of appetite, the HPA axis may respond to stress with a hypo-activation of the HPA axis and the (atypical) neurovegetative symptoms of increased appetite and weight gain [12]. In this line of thought, emotional eaters do not *have* the typical post-stress reduction of hunger, but instead have similar or higher feelings of hunger after stress (for support, see Fig. 2 in 13; see also [32]) (study [32] was conducted in patients with binge eating disorder, a condition that is closely associated with high emotional eating). A lowered HPA axis functioning in response to stress would also explain why emotional eaters are more receptive to the reinforcing value of food and use food as ‘self-medication’ to (temporarily) blunt the effects of stress and negative emotions (but see the meta-analysis of the affect regulation model by Haedt-Matt and Keel [33]).

Adverse rearing experiences early in life, particularly so when they involve parent-infant relationships, were, in some studies, shown to have lasting effects on stress-responsive neurological systems [34, 35]. And though high emotional eaters have also been observed to respond to acute stress with increases in cortisol [36, 37], Tomiyama et al. [38] found that women who experienced chronic stress (because they were caregivers of chronically ill children) had both higher scores on a scale for emotional eating *and* showed a dampened HPA axis activity in comparison with women without such chronic stress.

The hypothesis that high emotional eaters with a blunted HPA axis stress activity (blunted cortisol stress response) would show the highest food intake after stress was indeed conformed in a study by van Strien et al. [19] using as stressor the TSST (Trier Social Stress Test) [39]. Also in two other studies [40, 41], there was evidence that a blunted HPA axis stress response led to increased food intake. However, whether a blunted cortisol stress response is indeed a cause or rather a response to emotional eating is as yet unresolved [19]. A blunted cortisol stress response has also been conceived as being secondary to emotional eating, as a result of adaptive downregulation of the HPA-as in response to frequent overeating [18].

## Emotional Eating and External Eating

Emotional eating tends to co-occur with external eating (i.e. overeating in response to food-related cues such as the sight and smell of attractive food) [42]. This co-occurrence (*r* = .50) has been explained with the escape-of-self-awareness theory by Heatherton and Baumeister [43], where some people (e.g. emotional eaters) shift their attention away from their negative affect by narrowing it to the immediate (food) environment with, as outcome, external eating. In support, in an experiment by Slochower [44], negative emotions and food cues were shown to operate conjointly to elicit overeating in female students with obesity—the participants only overate in the high anxiety—high food salience condition, but not when the anxiety and/or the food salience was low. Similarly, in a functional neuro-imaging study in female chronic dieters, negative affect was associated with an increase in the reward value of appetizing food cues, as indicated by an increased activity in the orbitol frontal cortex (OFC) [45]. In a subsequent study, this increase was found to predict weight gain 6 months later [46].

Although emotional and external eating as measured by a self-report questionnaire often co-occur, they refer to independent constructs, as the items measuring emotional and external eating were shown to load on different factors [47], and the scales for emotional and external eating showed good discriminant validity in relation to other variables, such as depressive feelings [48, 49, 50•]. When Paans et al. [50•] used external eating as covariate in the analysis on emotional eating, and vice versa, results showed that the association of depression diagnosis and severity of depression with emotional eating remained significant, whereas the associations with external eating became non-significant, suggesting that ‘pure’ emotional eating but not ‘pure’ external eating is associated with depression ([50•], p. 41).

A key difference between the two eating styles is that external eating, in contrast to emotional overeating, has been considered an evolutionary adaptive response ([51] p. 361) which is based on the concept of thrifty genotype by Neel [52]. It is suggested that evolution favored genetic adaptations that allow humans to survive in periods of food shortages, including selective pressure to overeat whenever food is present (see further, [15]). In support, tendency towards external eating showed, in contrast to emotional eating, a high prevalence in children [53]. A further important difference between the two eating styles is that external eating may not be an ‘obese eating’ style, an eating style associated with a higher body mass index (BMI) or overweight. In various studies (references in [15], p. 385), overweight women showed similar levels of external eating as women with a normal body weight. In contrast, there is robust evidence that self-reported emotional eating is an ‘obese’ eating style, as it was repeatedly shown to be associated with overweight and prospective weight gain [54–56] also in interaction with negative life events [57] and short sleep duration (a marker of distress) [58].

## Emergence of Emotional Eating in Adolescence

Emotional eating is highly prevalent in (female) adults with overweight or obesity [15, 56]. In children, in contrast, the prevalence of emotional eating is, as already stated, very low [53, 59]. This suggests that most young children show the natural response of losing appetite in response to negative emotions or stress and that emotional eating emerges in the transition between childhood and adulthood: the period of adolescence (possibly as result of estrogen activation at puberty [60, 61] p 905). Two prospective studies in Dutch families with two adolescent children addressed the emergence of emotional eating in adolescence [62, 63].

In the first study (*n* = 279), the emergence of emotional eating was assessed in relation to the interaction between inadequate parenting practice of psychological control (e.g. ‘My father (mother) makes me feel guilty when I fail at school) and the dopaminergic (reward sensitivity) brain system, namely a polymorphism in the dopamine D2 receptor gene (*DRD2*) [62]. Carrying the A1 allele of the *DRD2* gene Taq1A polymorphism (rs1800497) is associated with reduced *DRD2* receptor availability in the brain [64]. *DRD2* genotype was found to moderate the association between maternal and paternal psychological control and prospective increase in emotional eating. Adolescent girls *and* boys showed an increase in emotional eating after 4 years in relation to high psychological control at baseline only if they carried at least one *DRD2* A1 allele.

In the second study, the emergence of emotional eating was assessed in relation to the interaction between depressive feelings and a serotonergic brain system, namely polymorphisms in the serotonon transporter (*5-HTT*) gene (*SLC6A4*) [63]. The short allele of the *5-HTTLPR* polymorphism in the serotonin transporter gene is associated with lower serotonin activity [65]. Lower levels of serotonin activity are associated with increased appetite and body weight [66]. The serotonin transporter gene indeed moderated the association between depressive feelings and increase in emotional eating after 4 years. In the youngest siblings (who had been 13 years at baseline) (*n* = 286), this was true for both the girls and the boys. In the oldest sibling (who had been 15 years at baseline) (*n* = 298), this was only true for the girls, possible because girls of this age have a higher prevalence of both depression and emotional eating (see further, van Strien et al. [63], or because of sex differences in the interactions with the *5-HTTLPR* polymorphism (see the review by Gressier et al. [67]).

In both studies, there were no main effects of the polymorphisms. Only in interaction with negative parenting or depressive feelings, they predicted future increases in emotional eating. When looking more closely at the interaction effects, they support Belsky’s et al. [68] framework of ‘plasicity genes’: the notion that ‘risk-genes’ are associated with increased sensitivity to the environment. Adolescents with at least one risk gene (at least one *DRD2* A1 allele) who grew up under the favorable rearing experience of not experiencing parental psychological control showed the lowest increase of emotional eating after 4 years and seemed to fare even better than adolescents carrying the A2/A2 genotype. The same held true for young adolescents (13 years) and the older adolescent girls (15 years) who combined carrying at least one *5-HTTLPR* short allele with low depressive feelings. The notion of flexibity genes also opens perspectives for prevention, namely that people with ‘risk genes’ may fare better when the environment is supportive. See for a similar conclusion and the relevance of the ‘differential susceptibility’ hypothesis for obesity the 2017 review by Molle et al. [69•].

## Emotional Eating as Mediator Between Depression and Obesity

Feeling depressed is normally associated with loss of appetite and subsequent weight loss. There exists, however, a subtype of depression that is characterized by the a-typical features of increased appetite and subsequent weight gain (DSM-5; American Psychiatric Association, [70]). Emotional eating has been considered a marker of this depression subtype [63] because it shares with this subtype the a-typical feature of increased appetite in response to distress such as feelings of depression (for support, see [50]). In the meta-analysis of Luppino et al. [71], depression was found to be associated with subsequent weight gain and obesity, and the question arose whether emotional eating is the missing link between depression and obesity or weight gain.

In various cross-sectional studies, emotional eating was indeed found to act as a mediator between depression and obesity [72–75]. In a further prospective study on the parents of the adolescents in the Van Strien et al. [62, 63] studies [76••], emotional eating acted as a mediator between maternal depression and weight gain after 5 years. Depressive symptoms were related to higher emotional eating; emotional eating also predicted greater increases in BMI independently of depression. No causal chain between depression, emotional eating, and weight gain was found in the fathers, possibly because both a-typical depression and emotional eating are less prevalent in men [76••].

It should be noted that most studies that found mediation effects for emotional eating have been carried out in countries that are located in northern latitudes, where a-typical depression is more common [75]. Exceptions are the study in females by Clum et al. [72] and the study in Spain by Van Strien et al. [75], which were both conducted after stressful life events. The study by Clum et al. [72] was conducted 1 year after the hurricane Katrina, in the Greater New Orleans area (30°00′ northern latitude), whereas massive unemployment after the 2008 banking crisis was an ego-threatening stressor in Spain (26°00′–44°00′ northern latitude) [75].

Depression and obesity are both common conditions with severe medical consequences and high costs for society [77, 78]. The finding that emotional eating is a mediator between the two suggests that a reduction of emotional eating may be an important treatment target for both obesity and (a-typical) depression.

## Matched Treatment of Obesity

The role of emotion regulation in childhood obesity and its implications for prevention and treatment of obesity has recently been reviewed by Aparicio et al. [79]. It was concluded that teaching emotion regulation skills could be an effective approach for treating obesity in children. For obese adults, where a recent systematic review found no evidence for a general deficit in emotion processing [79], I would instead suggest a matched treatment approach, an approach where the treatment is matched to the specific characteristics of the individual.

One of the reasons that most weight reduction programmes do not result in permanent weight loss for most individuals may be an absence of fit between treatment and individuals [80]. Overeating has different reasons for different persons. As already indicated, one person may overeat after a period of slimming when the cognitive resolve to eat less than desired is abandoned (for example, due to stress or negative emotions) (restrained eating), another may have the tendency to overeat when seeing or smelling delicious food (external eating), while a third eats too much when experiencing negative emotions (emotional eating). Each type of eating behaviour has its own aetiology, and each type requires its own type of treatment. When treatments are related to an individual type of eating behaviour, it can be expected that the obtained weight loss is more permanent [42, 81].

### The DEBQ

The three eating behaviours can be measured with the Dutch Eating Behavior Questionnaire (DEBQ [42, 81]). Though eating styles can also be measured with other instruments (references in Barrada et al. [47]), the DEBQ was the first instrument that covered the three eating styles with unidimensional scales [11]. The instrument is highly cited (> 2300 citations in Google Scholar) and has been translated in more than 15 languages (e.g. German [82], Chinese [83], Spanish [84]). The three scales have good reliability, dimensional validity [47] and predictive validity for, respectively, distress-induced food intake [85, 86], eating in response to food cues [87] and eating less than desired [88] and the DEBQ has been rated as ‘up to the mark’ or ‘good’ by the Dutch Committee on Tests and Testing (COTAN) on all EFPA (European Federation of Psychologists’ Association) criteria (e.g. norms, reliability (internal consistency, test-re-test) and validity (dimensional validity, construct validity and criterion validity) (COTAN, [89])).

### The DEBQ and Treatment

Treatment of people with a high degree of emotional eating (a scale score above the mean or higher compared to the closest norm group) should focus on a more adequate response to emotions, for example by teaching emotion regulation skills. In a pilot study of a group dialectical behaviour therapy (DBT), a cognitive behaviour therapy adapted for obese emotional eaters with modules on mindfulness (a nonjudgmental state of awareness of one’s thoughts, feelings or experiences in the here and now), emotion regulation and distress tolerance [90]. DBT appeared to be an effective intervention for reducing body weight and (eating-related) pathology (emotional eating and depression) at follow-up [90]. A further option for treatment may be mindfulness training (see the review by Warren et al. ([91], p. 277). Enhancing mindfulness was also shown to enhance the cortisol response to a stressor [92] and to change the cortisol awakening response and to reduce abdominal fat [93].

A high degree of external eating, unsupported by a high degree of emotional eating, points to a sensitivity to external food cues. Although mindfulness approaches were also shown to be effective for external eaters with obesity [91], the therapy could best focus on the sensitivity to food cues by means of behavioural methods such as stimulus control, cue exposure or food go/no-go training [81, 94].

In people with low scores on emotional or external eating, but with high scores on restrained eating, also the past history of body weight and weight fluctuation should be taken into account, as should be the tendency towards restrained breaking (disinhibition or failure of restraint) or bingeing. For example, underweight in combination with severe dieting may point to anorexia nervosa, while a history of large weight fluctuation in combination with severe dieting and tendencies towards restraint breaking may indicate bulimia nervosa. However, if a person has always been overweight, he or she may be better of accepting his or her heavy build than to continuously starving him or herself. In approaches such as Health at Every Size and Intuitive eating, the focus is on health and adaptation and getting into contact with feelings of hunger and satiety [95].

## Summary and Conclusion

Possible mechanisms of distress-induced emotional eating are poor interoceptive awareness, high alexithymia and a blunted HPA axis stress response (as indicated by a blunted cortisol stress response). Inadequate parenting and a high degree of depressive feelings may, in interaction with a genetic vulnerability, be associated with increases in emotional eating during adolescence. Emotional eating may also act as a mediator between depression and body mass index or weight gain. This finding is of interest for both obesity interventions and interventions for atypical depression. A matched treatment approach of obesity is suggested, an approach that matches the treatment to the specific eating style of the individual. Matched treatment of obesity may provide a new pathway to more permanent weight loss or maintenance of body weight [7].
